# Attenuation of Vaccinia Virus

**Published:** 2015

**Authors:** S. N. Yakubitskiy, I. V. Kolosova, R. A. Maksyutov, S. N. Shchelkunov

**Affiliations:** State Research Center of Virology and Biotechnology “Vector”, Koltsovo, Novosibirsk region, Russia; Institute of Cytology and Genetics, Siberian Branch, Russian Academy of Sciences, Novosibirsk, Russia

**Keywords:** vaccinia virus, virulence genes, vaccine, attenuation, protection

## Abstract

Since 1980, in the post-smallpox vaccination era the human population has become increasingly susceptible compared to a generation ago to not only the variola (smallpox) virus, but also other zoonotic orthopoxviruses. The need for safer vaccines against orthopoxviruses is even greater now. The Lister vaccine strain (LIVP) of vaccinia virus was used as a parental virus for generating a recombinant 1421ABJCN clone defective in five virulence genes encoding hemagglutinin (*A56R*), the IFN-γ-binding protein (*B8R*), thymidine kinase (*J2R*), the complement-binding protein (*C3L*), and the Bcl-2-like inhibitor of apoptosis (*N1L*). We found that disruption of these loci does not affect replication in mammalian cell cultures. The isogenic recombinant strain 1421ABJCN exhibits a reduced inflammatory response and attenuated neurovirulence relative to LIVP. Virus titers of 1421ABJCN were 3 lg lower versus the parent VACV LIVP when administered by the intracerebral route in new-born mice. In a subcutaneous mouse model, 1421ABJCN displayed levels of VACV-neutralizing antibodies comparable to those of LIVP and conferred protective immunity against lethal challenge by the ectromelia virus. The VACV mutant holds promise as a safe live vaccine strain for preventing smallpox and other orthopoxvirus infections.

## INTRODUCTION

Smallpox was declared eradicated in 1980 by the World Health Organization, after which human vaccination against smallpox was halted [1]. This decision was mainly due to a variety of adverse events, ranging in severity from benign to lethal [1, 2].

Following its termination, smallpox immunity in the human population has been decreasing. Naive individuals become susceptible to not only variola (smallpox) virus (VARV), but also related orthopoxviruses, which are mainly maintained in the rodent population as an animal reservoir. [3]. Humans and animals can be infected by the monkeypox virus (MPXV) and cowpox virus (CPXV). A wide distribution in the human population is likely to allow orthopoxviruses to adapt to the human immune system and lead to new virus strains infectious for humans [3, 4]. Recently, an increased rate of zoonotic poxvirus outbreaks has been reported worldwide [3], the concerns about potential bioterrorist attacks with VARV have been discussed [5].

The only way to protect against the growing threat posed by orthopoxviruses is vaccination [1, 2]. Immune deficiency in humans for the last decades has led to a situation whereby classic live vaccinia-virus-based vaccines cannot be efficiently used for severe, associated complications that rarely ensued during the smallpox vaccination era. Consequently, there is an urgent need for orthopox vaccine candidates with a perfect safety record and high immunogenicity, as compared to those used in the past.

First, attenuated VACV strains were produced through multiple passages on chicken embryo fibroblasts (strain MVA) [6] or cell cultures of rabbit kidney (strain LC16m8) [7]. Attenuation was accompanied by spontaneous, extended deletions and other mutations in viral genes implicated in virulence, replication capacity, and host-range [5].

With the advent of genetic engineering tools, liveattenuated vaccine strains can be tailored to the particular virus variant by inserting, deleting, or disrupting genes of choice [8]. Attenuated strains generated by the deletion of virulence genes retain replication competence but are no longer pathogenic. Promising vaccine candidates are recombinant attenuated VACV strains, capable of eliciting protective antibodies comparable with the smallpox vaccine, but with much reduced post-vaccination side-effects.

Our expertise in complete genome sequencing of orthopoxvirus species and strains pathogenic for man [9-12], targeted mutagenesis [15, 16], and knowledge of the functions of the genes of these viruses [2, 13, 14] allowed us to elaborate and implement a novel approach towards generating attenuated VACVs. This approach involves the targeted excision or disruption of a virulence gene, without affecting viral replication and host-range.

The objective of this work was twofold: (i) to engineer a VACV variant with a knockout in 5 virulence genes, which could be used as a promising live-attenuated vaccine candidate against smallpox and other orthopoxvirus-related diseases in humans; and (ii) to examine its biological characteristics. The isogenic VACV strain, designated 1421ABJCN, was compared to its parental strain LIVP (clonal variant) in terms of viral propagation in cell cultures, pathogenicity in mouse and rabbit models, protective immunogenicity, and protection of experimental animals against a lethal challenge with a virulent orthopoxvirus. These characteristics are carefully considered in pre-clinical trials of new vaccines against orthopoxvirus infection [17].

## EXPERIMENTAL

**Bacteria, viruses, cell cultures.**

In this study, we used the *Escherichia coli *strains JM109 and XL2-blue, the VACV strain LIVP (derived from the Lister strain background obtained at the Institute for antivirals, Moscow) and the ectromelia virus (ECTV) strain K-1 from the collection of SRC VB Vector, passaged on cultures of African green monkey kidney cells CV-1, and Vero and 4647 from the cell culture collection of SRC VB Vector.

**Integrating plasmids**

**Fig. 1:**
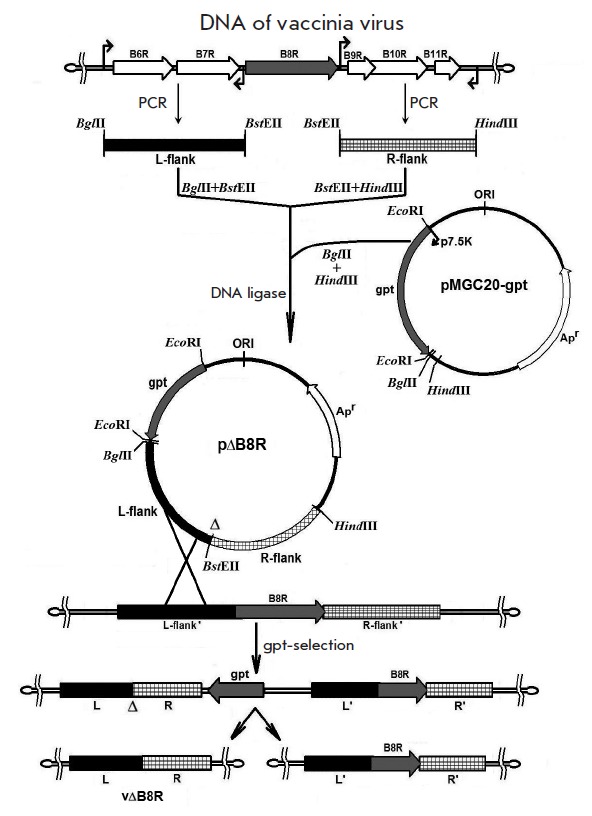
Schematic diagram illustrating the generation of VACV B8R mutants (see text for details)

**Fig. 2:**
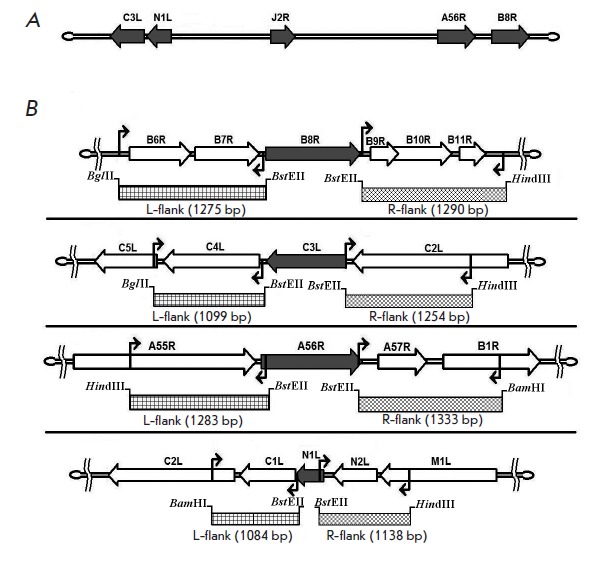
Schematic diagram illustrating the amplification of PCR fragments flanking the deleted sequences in the VACV genome. A. Map of the VACV genome with the location of the *C3L, N1L, J2R, A56R, *and *B8R *virulence genes (indicated with arrows); B. Schematic of amplified regions (shaded area) flanking a sequence stretch upstream (L-flank) and downstream (R-flank) of the deleted genes (grey block arrows). The zigzag arrows stand for the position of primer pairs so that a deletion in one gene does not affect the adjacent genes and amplified DNA fragments are flanked by the restriction sites

Integrating plasmids used for the deletion of target genes in the VACV genome were derived from the backbone vector pMGC20-gpt carrying the selective marker *E. coli gpt*-gene under the control of the VACV 7.5K promoter [16] (*Fig. 1*). Upstream (L-flank) and downstream (R-flank) DNA fragments flanking the gene to be deleted were amplified by PCR from the vaccinia virus strain LIVP, using primer pairs with restriction sites (*Fig. 1*, *Fig. 2*). Both flanking sequences were digested and inserted into the plasmid pMGC20- gpt upstream and downstream of each gene (*Fig. 2*). Correct construction of pΔB8R, pΔC3L, pΔA56R, and pΔN1L was confirmed by restriction profiling and sequencing.

The plasmid pΔTK carries a MCS sequence which inactivates the thymidine kinase gene (*J2R*) through to an in-frame translation disruption [18].

**Generation of VACV strains with deleted/disrupted virulence genes**

VACV was propagated on a CV-1 cell culture in a DMEM medium supplemented with 2% fetal bovine serum. All mutant VACV variants were engineered using the transient dominant selection strategy [16]. Following 4-5 passages in the selective medium (before the onset of cytopathic effects), the virus was plaque-purified using an agarose overlay [19] and replated in a non-selective medium for secondary cloning. DNA of plaque-purified clones of recombinant VACVs was extracted as described [15]. After PCR verification, one clone of the 2-3 plaque-purified clones was titered by the plaque assay method on CV-1 [19].

**PCR analysis of recombinant VACV variants**

Obtained VACV clones were verified for the desired insertions/deletions by PCR with the corresponding primers:

∆B8R: 5’-TCACAAATATGATGGTGATGAGCGA-3’ 5’-CGTGATATACCCTAGCCATAGGCAT-3’.

∆C3L: 5’-TCGCGCTTTACATTCTCGAATCT-3’ 5’-TGTTCGTGTGTTCTTGCGGTGA-3’.

∆A56R: 5’-GTGGTATGGGACACCACAAATCCAA-3’ 5’-ATTAAACATTCCTAGAATTAATCCCGCTC-3’.

∆N1L: 5’-GGGTTGGATCCTTTACACATAGATCTACTACAGGCGGAACA- 3’

5’-GGGAAAGCTTAATTTGTGAAGATGCCATGTACTACGCT- 3’.

J2R-MCS: 5’-ATATGTTCTTCATGCCTAAACGA-3’ 5’-ATGAAGGAGCAAAAGGTTGTAAC-3’.

PCR was carried out in 0.2 ml tubes (Applied Biosystems, USA) on a GeneAmp PCR System 9700 thermal cycler (Applied Biosystems, USA). The reaction mix contained SE buffer for Taq-DNA polymerase, NTPs and Taq-DNA polymerase (SibEnzim, Russia), and sterile deionized water. The thermal profile consisted of an initial denaturation step at 940C for 1.5 min, followed by 20 cycles of 940C for 20 s, 550C for 30 s, 720C for 1 min, and ending with a final elongation step at 720C for 5 min. PCR fragments were stored at 40C until needed.

**Growth curves**

To evaluate viral replication kinetics, the parental clone 14 of VACV LIVP and mutant VACV strains with inactivated virulence genes were seeded onto 90–100% confluent monolayers of CV-1 or Vero in 6 well plates at a multiplicity of infection (m.o.i) of 0.1 pfu/cell. Viral titers were determined by plaque assay at time points 0, 24, 48, and 72 h post infection.

**Harvest and purification of mutant VACVs**

A monolayer of cell line 4647, which is recommended for variola virus production [20], grown on tissueculture flasks with a growth area of 175 cm^2^ (volume 650 ml), was infected with VACV at a multiplicity of infection of 1.0 pfu/cell. Cells were incubated for 48 h at 37°C until the cytopathic effect was evident throughout the culture. A total of 80% of the maintenance medium was removed, and the remaining 20% medium (10 ml) was lysed by three cycles of freeze-thawing, followed by ultrasonic treatment with a MSE 500 disintegrator (22 kHz, 2-3 times for 10-15 s). Cell debris was removed by centrifugation (4,000 g for 10 min). The supernatant was centrifuged at 30,000 g for 1.5 h. The pellet of virus was re-suspended in 4 ml isotonic saline. Infection titers were determined by plaque assay on 4647 monolayer cells.

**Animals**

Depending of the experiment, Balb/c female mice weighing 14–16 g (aged 4–5 wk) or 1-2-day-old suckling mice of the same strain weighing 5–6 g were used. The animals were placed in groups of ten. We also used Chinchilla rabbits weighing 2.5–3 kg. All animals were obtained from the animal facility of SRC VB Vector. The use and care of animals followed the guidelines for the experimental use of animals. Animal care procedures were approved by the Animal Ethical Committee of SRC VB Vector.

**Assessment of mutant VACV virulence**

To assess the virulence properties of the genetically engineered VACV strains, 1-2-day-old suckling mice received an intracerebral inoculation of recombinant VACV 1421ABJCN or the parental VACV LIVP clone 14, diluted in isotonic saline at a dose of 102 pfu/0.01 ml per mice. Mock-infected controls were inoculated with isotonic saline. Infected animals were examined for death on a daily basis over an 8-day period.

To determine if recombinant VACV strains can replicate and propagate in the brain cells of suckling mice inoculated by the same route, mice were euthanized by cervical dislocation on day 3 post infection and brain tissue was aseptically sampled. Samples from each group were pooled to prepare a 10% suspension in a DMEM medium as previously described [21]. Infection titers were determined by plaque assay on 4647 monolayer cells.

Testing for virulence in rabbits was performed by intradermal injection in a shaved area on the lateral aspect of each flank. Virus suspensions were serially 10- fold diluted in isotonic saline to give 102–107 pfu/0.05 ml, followed by intradermal inoculation in the volume as described above. Each dilution was injected at two separate sites across a shaved area of each rabbit: one flank for VACV 1421ABJCN and the other flank for the parental VACV LIVP. The animals were monitored daily for up to 14 days for the occurrence and disappearance of skin lesions, depending on titer and virus strain.

**Immunogenicity**

The presence of specific antibodies was evaluated by the neutralization activity of the serum of 4- to 5-week old mice infected with 1421ABJCN or LIVP in a dose range of 106, 107 or 108 pfu/0.1 ml per mice. Control mice were sham-inoculated with isotonic saline. The immunization was carried out twice at a 28-day interval. On day 28 post second immunization (day 56 post first immunization), ether-anesthetized mice were bled via the retrobulbar venous plexus, blood was allowed to clot overnight at 40C overnight, and the sera separated from cellular constituents by centrifugation at 5,000 g for 10 min. The sera from each group were pooled and heated at 560C for 30 min for the inactivation of complement. Virus neutralization titers were determined on a 4647 cell culture as described [22].

**Protective immunity**

The ability of engineered VACV strains to protect against a lethal orthopoxvirus challenge was assessed in 4- to 5-week old mice following the immunization procedure as described (see Immunogenicity section). On day 28 post second immunization, lightly anesthetized mice were intranasally infected with highly pathogenic ECTV at a dose of 10 LD_50_/0.02 ml per mice as previously reported [23]. Survival and mortality of the inoculated mice were monitored for 14 days.

**Statistical analysis**

Experimental data were analyzed by Student’s t-test using the Origin Professional 8.1.10.86 software. P < 0.05 was considered statistically significant [24].

## RESULTS

**Generation of vaccinia virus clones**

Before plasmid integration, individual clones of LIVP VACV were isolated by serial plaque-purifications us ing agarose overlay [19] to ensure strain homogeneity. Five candidate VACV clones were picked for subsequent genomic DNA extraction and *Hind*III restriction profiling. Clone 14, which was consistent with the restriction pattern of the parental LIVP VACV, was selected.

**VACV clones with targeted disruption of virulence genes**

The genes required for virulence, the interferon-γ (IFNγ) binding protein (*B8R*), secreted complementbinding protein (*C3L*), hemagglutinin (*A56R*), endogenous apoptotic inhibitor (*N1L*), and thymidine kinase (*J2R*) were sequentially inactivated (*Fig. 2A*). The targeting plasmids p∆B8R, p∆C3L, p∆A56R, p∆N1L, and p∆TK were used to attempt plasmid integration and gene disruption.

CV-1 confluent cells were infected with VACV LIVP clone 14 and transfected with recombinant p∆B8R plasmid under gpt-selection. Single-crossover integration of the targeting plasmid into the viral genome produced a viral DNA genome carrying a gpt selection marker, a continuous stretch of viral DNA with targeted deletion of the gene, and the same stretch of viral DNA without deletion. This recombinant DNA segment can only be retained under selection conditions [15, 16]. Due to rapid deletion of *gpt *gene upon removal of selection and high frequency of intramolecular recombination within the R-R’ region, recombinant vΔB8R was obtained (*Fig. 1*). Candidate clones of this variant were identified by PCR (see Experimental section, data not shown).

**Fig. 3:**
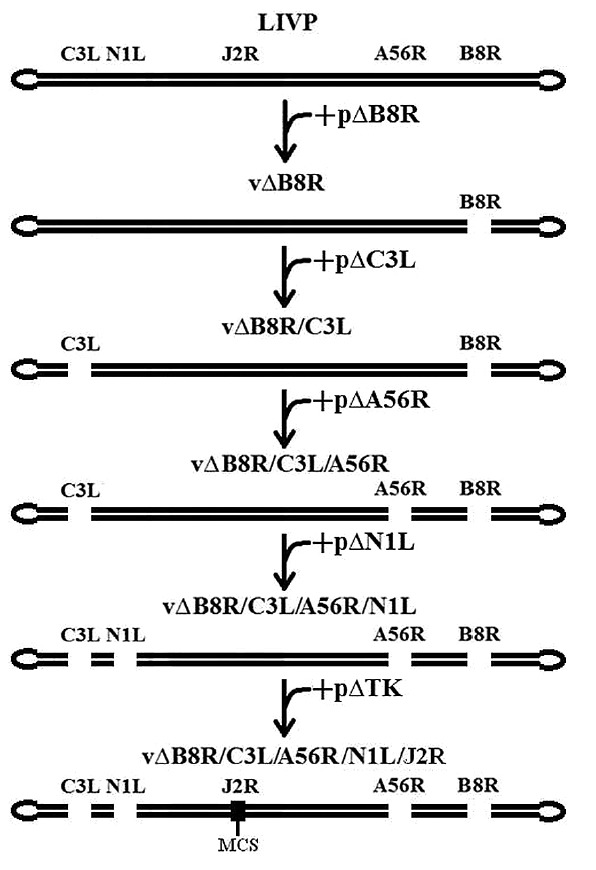
The strategy to introduce mutations in each of the VACV virulence genes. The order of sequential inactivation with plasmids targeting the genomic sites of choice is shown

One B8R-negative clone was further subjected to deletion of the *C3L *gene to produce a VACV strain negative for *B8R *and *C3L*, followed by sequential disruption of the other three loci *A56R*, *N1L*, and *J2R* (*Fig. 3*). The final strain with disrupted *B8R*, *C3L*, *A56R*,* N1L*, and *J2R* genes was designated 1421ABJCN.

**Fig. 4:**
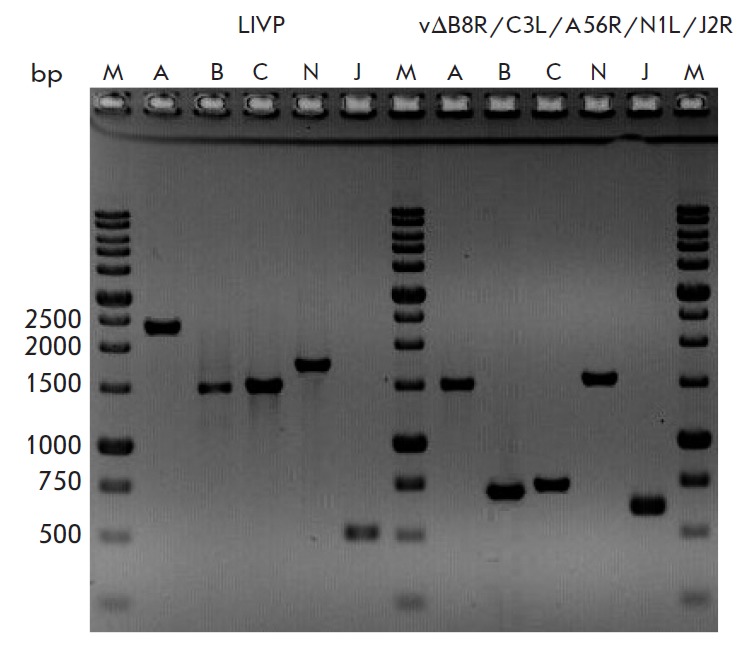
Verification of deletions/insertions by PCR. PCR products formed from DNA of the parent clone 14 VACV LIVP and 1421ABJCN with disrupted virulence genes (see text for details). M – molecular size marker, the lengths are given on the left . Lanes A, B, C, N, J contain VACV DNA fragments amplified from the genes *A56R, B8R, C3L, N1L, *and *J2R*, respectively

The expected deletions/insertions were verified by PCR protocols targeting the mutated genes (see Experimental section). *Fig. 4* shows the PCR analysis of the 1421ABJCN strain with disrupted genes. The fullsize PCR products of the genes *A56R*, *B8R*, *C3L*, *N1L*, and *J2R*, amplified from the parental clone LIVP, were 2366, 1555, 1542, 1784, and 512 bp, respectively. PCR fragments appropriate to the deletion variant genes of ∆*A56R*, ∆*B8R*, ∆*C3L*, and ∆*N1L *and the insertional variant *J2R-MCS *of 1421ABJCN were 1425, 737, 751, 1431, and 617 bp, respectively, which was consistent with the expected sizes.

**Growth curves for mutant VACV in mammalian cell cultures**

**Fig. 5:**
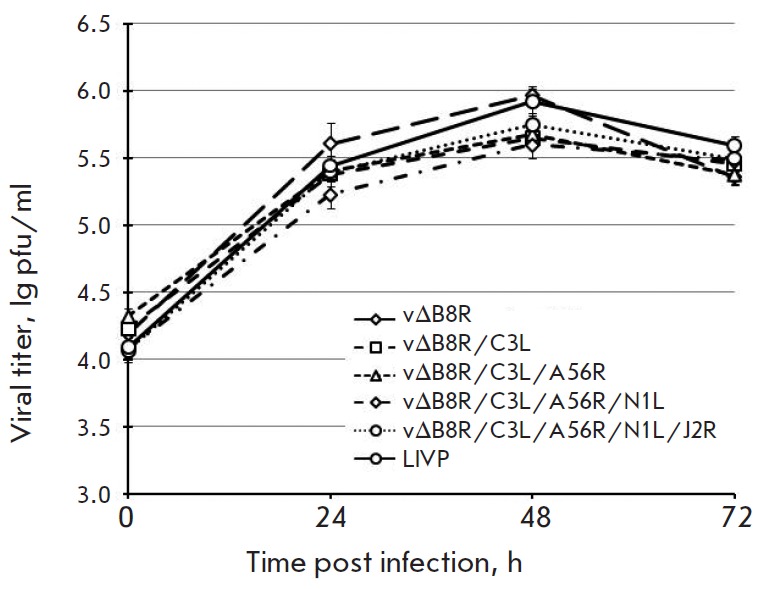
Growth curves for VACV mutants in CV-1 cells

The highly attenuated vaccine strain must have permissive replication in mammalian culture cells. For this reason, the engineered VACV recombinants were characterized for growth kinetics in CV-1, Vero, and 4647 cells. To analyze viral replication titers, the cell cultures were infected with VACV variants with a different combination of disrupted genes. Growth curves of the recombinant viruses in CV-1 cells (*Fig. 5*) showed no significant differences in replication properties, as well as when compared to the parental VACV LIVP. Similar findings were observed for Vero and 4647 cells.

We found that recombinant VACV variants displayed robust growth and retained a stable genotype over 10 passages in CV-1 and 4647 cells.

**Neurovirulence**

**Fig. 6:**
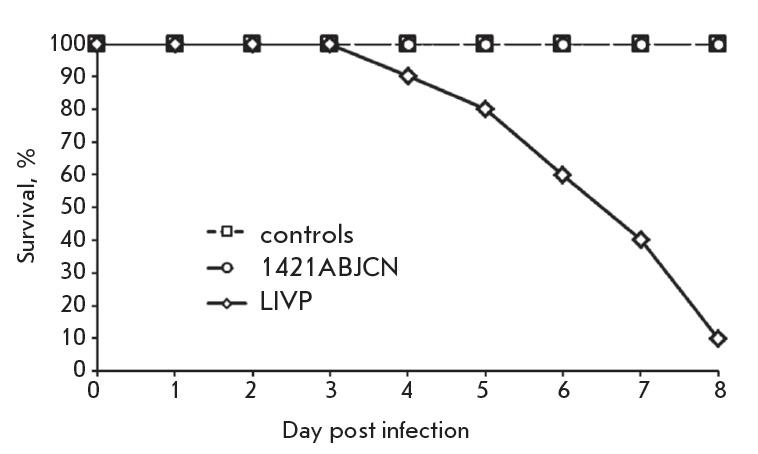
Time-course of mortality following intracerebral infection of newborn mice with 1421ABJCN and LIVP VACV

Lethality after intracerebral challenge was studied in newborn mice observed for 8 days after inoculation. Mice infected with VACV LIVP at a dose of 102 pfu/ mice started dying from day 4, with mortality reaching 90% by day 8. Mice inoculated with VACV 1421ABJCN at the same dose showed no mortality (*Fig. 6*).

In addition, viral titters were determined in the brain of suckling mice on day 3 post infection. The growth characteristics of 1421ABJCN (virus titer ± SD: 2.78 ± 0.66 lg pfu/g of organ) were significantly different from those of the parental VACV LIVP (6.12 ± 0.20 lg pfu/g of organ).

**Pathogenicity for rabbits**

Pathogenecity studies were performed by bilateral shaving of both flanks, followed by intradermal inoculation of virus for edematous and necrotic lesions to occur. VACV 1421ABJCN caused an inflammatory response at a dose higher than 105 pfu per injection, whereas the parental VACV LIVP induced swelling at a dose of 102 pfu/injection (Table). Swelling and necrotic plaques of 1421ABJCN were less severe than those of VACV LIVP. The lesions induced by 1421ABJCN completely disappeared by day 9 post inoculation versus 14 days for LIVP lesions to heal.

**Table:** Edematous and necrotic lesions in a rabbit model of intradermal infection with VACV strains

Day post infection	Viral titter, pfu/injection											
	1421ABJCN						LIVP					
	10^2^	10^3^	10^4^	10^5^	10^6^	10^7^	10^2^	10^3^	10^4^	10^5^	10^6^	10^7^
1	-	-	-	-	S	S	-	-	-	S	S	S
	-	-	-	S	S	S	-	-	-	S	S	S
2	-	-	-	-	S	S	-	-	S	S	S	S/N
	-	-	-	S	S	S	-	-	S	S	S	S/N
3	-	-	-	-	S	S/N	S	S	S/N	S/N	S/N	S/N
	-	-	-	S	S/N	S/N	S	S	S/N	S/N	S/N	S/N
6	-	-	-	-	N	N	-	-	S/N	S/N	S/N	S/N
	-	-	-	-	N	N	-	-	S/N	S/N	S/N	S/N
7	-	-	-	-	-	N	-	-	S/N	S/N	S/N	S/N
	-	-	-	-	-	N	-	-	S/N	S/N	S/N	S/N
9	-	-	-	-	-	N	-	-	N	S/N	S/N	S/N
	-	-	-	-	-	N	-	-	-	-	S/N	S/N
11	-	-	-	-	-	-	-	-	-	N	N	S/N
	-	-	-	-	-	-	-	-	-	-	N	S/N
13	-	-	-	-	-	-	-	-	-	N	N	N
	-	-	-	-	-	-	-	-	-	-	-	N
14	-	-	-	-	-	-	-	-	-	-	-	N
	-	-	-	-	-	-	-	-	-	-	-	-

S – swelling, N – necrosis. Skin lesions were observed in two replicates for each virus dose

**Immunogenicity**

**Fig. 7:**
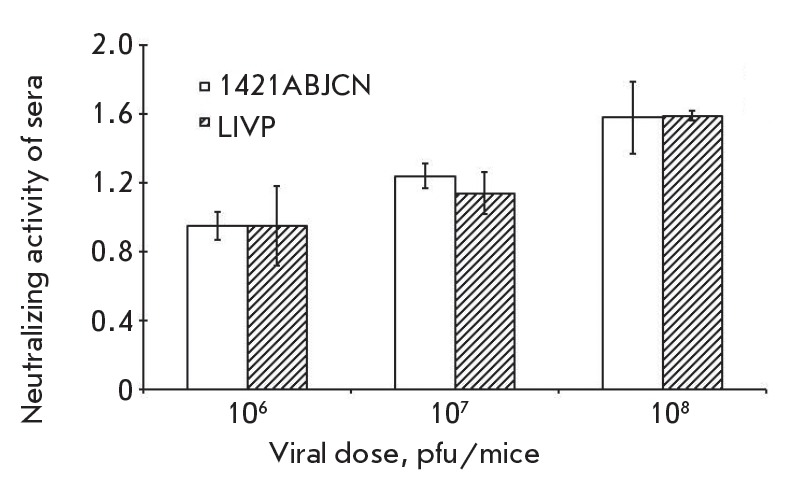
Levels of serum-neutralizing activity to VACV following double subcutaneous immunization of mice with 1421ABJCN and LIVP at different doses. Neutralizing titers were expressed in -log10 of the highest dilution which gives 50% neutralization of VACV ± standard deviation

The ability of VACV variants to elicit virus-neutralizing antibodies was examined in mice on day 28 post second immunization. Mice were infected at doses of 106, 107 or 108 pfu/mice. *Fig. 7* demonstrates that recombinant 1421ABJCN induces serum levels of virus-neutralizing antibodies comparable with the parental VACV LIVP. These findings suggest that the attenuated VACV variant has the same immunogenic properties as VACV LIVP. Sera of mock-infected controls had no detectable neutralizing antibodies to VACV.

**Protective properties of VACV variants**

**Fig. 8:**
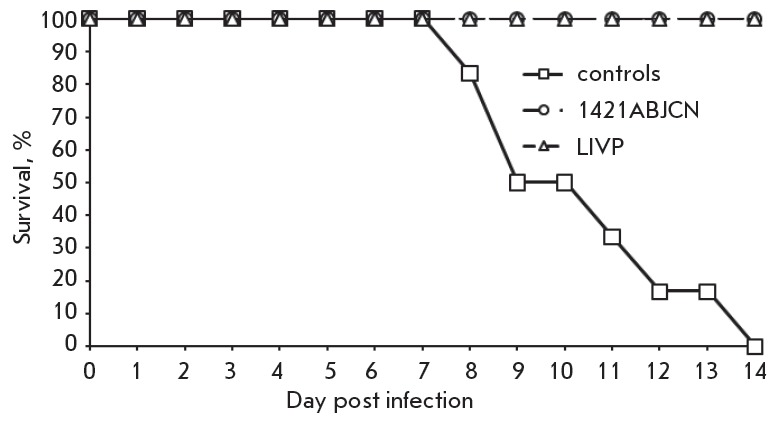
Time-course of mortality after double subcutaneous immunization of mice with 1421ABJCN or LIVP VACV at a dose of 10^6^ pfu/mouse, followed by challenge with ECTV at a dose of 10 LD_50_/mouse

To examine the protective efficacy of VACV variants against lethal challenge, mice were intradermally immunized twice at different doses (106, 107 or 108 pfu/ mice), followed by intranasal ECTV administration at a dose of 10 LD_50_/mice. Infected mice were examined for death over a 2-week period. Immunization with both 1421ABJCN and VACV LIVP at all doses conferred 100% protection (*Fig. 8*).

## DISCUSSION

The genus *Orthopoxvirus *of the family *Poxviridae *includes species pathogenic to humans such as VARV, MPXV, CPXV, and VACV. VARV is a dangerous human pathogen that has caused millions of human casualties in the past [1]. VARV has evolved to become an exclusive anthroponotic agent. Owing to the absence of other natural reservoirs (susceptible hosts) and the development of a smallpox vaccine with vaccinia virus as the active constituent, smallpox was globally eradicated [1, 2].

Following the termination of the program of routine vaccination since 1980, the number of humans susceptible to smallpox and other orthopoxviruses has increased due to lack of seroprevalence [4]. This situation increases the risk of infection and further transmission of orthopoxviruses in the human population, which could re-emerge as novel pathogens, spreading efficiently from human-to-human [3].

To construct a highly attenuated VACV variant for use as an orthopoxvirus vaccine and/or oncolytic virus, we sequentially deleted/disrupted the genes responsible for viral virulence. A literature search allowed us to identify putative virulence genes of VACV suitable for targeting: *B8R*, *C3L*, *A56R*, *N1L*, and *J2R*.

The *B8R *gene of VACV encodes a secreted glycoprotein in the form of a homodimer in infected cells at the early stage of infection. This protein is homologue to the extracellular domain of the IFN-γ receptor, capable of inhibiting IFN-γ activity in various mammalian models [25]. Deleting *B8R *gene results in attenuation with regard to the wild-type strain in mouse infection studies [26] and mild disease even at high doses [27].

The *C3L *gene codes for a secreted complementbinding protein (CBP) in infected cells at the early stage of infection [28] and inactivates complement through interaction with C3b and C4b [14, 29]. CBP suppresses the inflammatory response [30] and antibody- dependent neutralization of VACV virions aided by complement proteins [31]. In animal experiments, mutant VACV strains negative for CBP displayed reduced virulence [14, 31].

The early/late gene *A56R *encodes a surface hemagglutinin protein that mediates viral attachment to host cells, inhibits fusion of infected cells, and promotes proteolytic activation of infectivity. Deletion of the *A56R* gene from the NYCBH VACV strain showed a 40-fold decrease in LD_50_ versus the parental strain when given to mice by the intracerebral and intranasal routes [32]. Inactivation of the HA gene of the vaccinia virus strain WR leads to significant attenuation [33].

The VACV *N1L *gene encodes an intracellular homodimer with early/late expression [34]. It is non-essential for replication *in vitro*, but it plays an important role in virulence *in vivo*. The protein N1L belongs to the family of Bcl-2-like proteins [35], inhibits apoptosis, and regulates the function of the nuclear factor kappa B pathway [36].

The early VACV *J2R *gene expresses a viral thymidine kinase. *J2R *mutant viruses exhibit reduced virulence* in vivo *[37, 38].

*Fig. 1* and *Fig. 2* show a schematic diagram for target inactivation of the virulence genes to generate the expected VACV recombinants. Deletions/insertions were sequentially introduced into the target genes a VACV LIVP clone 14 (*Fig. 3*) and verified by PCR analysis (*Fig. 4*) and sequencing.

Because we were concerned about a live, highly attenuated strain without replication deficiency in mammalian cell cultures, we sought to determine replication properties for mutant viruses on CV-1, Vero, and 4647 cells. Growth curves were performed, and the results (*Fig. 5*) indicate that VACV variants defective in one or more of the virulence genes replicated to titers comparable to those of VACV LIVP in all cell lines. These findings demonstrate that mutations in these genomic loci did not alter viral replication in mammalian cell cultures, which are needed for the manufacturing of live vaccines.

The neurological complications following vaccinia virus vaccination seem to be associated with viral infection in the brain, followed by encephalitis. The hallmark of the VACV vaccine strain is neurovirulence observed in intracerebrally infected mice [39, 40]. The vaccine strain VACV LIVP at a dose of 102 pfu/mice caused mortality in 90% of the mice by day 8 post inoculation, whereas the descendant strain 1421ABJCN had no virulence in an intracerebral mice model at the same dose (*Fig. 6*). The titer of the recombinant VACV 1421ABJCN was a 1,000-fold lower than that of the parental strain VACV LIVP in the brain of suckling mice on day 3 post infection.

In a rabbit model of intradermal infection, VACV 1421ABJCN was at least 2 log10 less virulent than the parent strain VACV LIVP (Table).

Both viruses elicited comparable levels of neutralizing antibodies when administered subcutaneously to mice (*Fig. 7*) and conferred complete protection against challenge with highly virulent ECTV (10 LD_50_/mice) even at the lowest used immunization dose (106 pfu/mice ) (*Fig. 8*).

## CONCLUSIONS

We constructed a genetically engineered, replication- competent in mammalian cultured cells, VACV 1421ABJCN with targeted inactivation of 5 virulence genes. Our results show that VACV 1421ABJCN vaccination leads to reduced reactogenic properties and neurovirulence versus the parent VACV LIVP that is currently used for human vaccination in the Russian Federation. Besides the attenuated phenotype, VACV 1421ABJCN is immunogenic and protective as LIVP. This virus holds promise for use as a next-generation orthopoxvirus vaccine strain and could serve as a safe vector for recombinant polyvalent vaccines and/or oncolytic viruses.

## Abbreviations

PFU: plaque forming units
VARV: variola virus
VACV: vaccinia virus
CPXV: cowpox virus
ECTV: ectromelia virus
IFN: interferon
CBP: complement binding protein
LIVP: a strain L-IVP of vaccinia virus
MCS: multiple cloning site
MPA: mycophenolic acid
PCR: polymerase chain reaction
CPE: cytopathic effect
gpt: xanthine-guanine phosphoribosyl transferase gene
